# Association Between Joint Hypermobility and Pelvic Organ Prolapse: A Hospital‐Based Cross‐Sectional Study

**DOI:** 10.1002/hsr2.73469

**Published:** 2026-11-03

**Authors:** Zekra Ali Aziz

**Affiliations:** ^1^ Department of Prosthetics and Orthotics, Technical Health and Medical College Erbil Polytechnic University Erbil Kurdistan Region Iraq

**Keywords:** Beighton score, connective tissue, epidemiology, joint hypermobility, pelvic floor dysfunction, pelvic organ prolapse

## Abstract

**Background and Aims:**

Pelvic organ prolapse (POP) is a prevalent disorder of the pelvic floor with multifactorial etiology. Generalized joint hypermobility (JHM) is a sign of systemic collagen laxity. This study investigated the association between generalized JHM and POP and examined other clinical correlates including parity, body mass index (BMI), heavy occupational lifting, and family history in Middle Eastern females.

**Methods:**

This hospital‐based cross‐sectional study evaluated 812 non‐pregnant women aged ≥ 20 years attending an outpatient gynecology clinic at Erbil Maternity Teaching Hospital between November 2021 and April 2024. POP was staged using the standardized Pelvic Organ Prolapse Quantification (POP‐Q) system. Generalized JHM was defined as a Beighton score ≥ 4/9. Multivariable logistic regression and correlation analyses assessed independent correlates and severity trends.

**Results:**

Among 812 participants, 444 had POP (54.7%) and 368 served as non‐POP controls (45.3%). JHM was present in 67.6% (300/444) of POP cases versus 10.9% (40/368) of controls (unadjusted OR 17.08, 95% CI: 11.64–25.07; *p* < 0.001). In multivariable logistic regression, JHM remained strongly associated with POP (adjusted OR [aOR] 28.11, 95% CI: 17.21–45.91; *p* < 0.001). Family history of POP (aOR 9.69, 95% CI: 5.58–16.83; *p* < 0.001), heavy lifting (aOR 6.48, 95% CI: 4.05–10.37; *p* < 0.001), and parity (per birth aOR 1.17, 95% CI: 1.08–1.27; *p* < 0.001) were also independently associated with POP. Beighton scores increased significantly across advancing POP‐Q severity stages (Kruskal–Wallis *p* < 0.001; Spearman's ρ = 0.30, *p* < 0.001).

**Conclusion:**

Generalized joint hypermobility is strongly and independently associated with pelvic organ prolapse, with Beighton scores showing a positive correlation across prolapse severity stages. While joint hypermobility screening represents a simple clinical examination tool, prospective longitudinal studies are needed to determine whether such screening can improve risk stratification or guide preventive pelvic floor care.

## 1 Introduction

Pelvic organ prolapse (POP) is a major public health concern affecting millions of women all over the world, leading to decreased quality of life, physical discomfort, urinary and bowel dysfunction, and sexual morbidity [1]. Classic predisposing factors are well established [1, 2], including advancing age, high parity, vaginal delivery, chronic intra‐abdominal pressure, and elevated body mass index (BMI). Growing attention has been paid to intrinsic matrix biology and altered connective tissue composition as pivotal determinants of pelvic floor integrity [3, 4, 5, 6].

The endopelvic fascia, uterosacral‐cardinal ligament complex, and muscularis of the vagina are important for the mechanical stability of the pelvic floor. The microstructural changes in extracellular matrix (ECM) remodeling, such as abnormal collagen fiber arrangement, a changed ratio of type I to type III collagen, and dynamic elastin degradation, have severely affected the tensile strength of pelvic support structures [4, 5, 6]. Generalized joint hypermobility (JHM) is a physical examination finding characterized by increased range of motion in multiple joints [7, 8]. While JHM can occur as a feature of syndromic conditions such as hypermobile Ehlers–Danlos syndrome (hEDS) or hypermobility spectrum disorder (HSD), in general observational settings, it is predominantly evaluated as an isolated clinical sign reflecting connective tissue laxity rather than a clinical syndrome diagnosis [7, 9].

Systematic reviews and meta‐analyses have shown a significant association between JHM and POP [7], but single‐center observational studies have sometimes shown conflicting results. These differences are largely due to different phenotyping criteria, age‐related decline in joint flexibility, lack of consistent definitions of the control group and poor control of confounding factors such as parity and heavy lifting. Moreover, there is little evidence regarding the strength of this association in Middle Eastern populations.

The present study aimed to evaluate the cross‐sectional association between generalized JHM and clinical POP in a large cohort of women in Erbil, Iraq. We also investigated whether JHM was independently associated with POP after adjusting for potential confounders including age, BMI, parity, heavy lifting, and family history.

## 2 Materials and Methods

### 2.1 Study Design and Setting

This hospital‐based cross‐sectional study was conducted in the outpatient gynecology clinics of Erbil Maternity Teaching Hospital, a major tertiary care academic hospital, Erbil, Kurdistan region, Iraq. Data were collected between November 2021 and April 2024. The protocol was reviewed and approved by the Scientific Research Ethics Committee of Erbil Polytechnic University (Approval No. 24/0027 HRE). The study was carried out in accordance with the ethical principles of the Declaration of Helsinki. Prior to participation, all subjects received a detailed description of the study aims, clinical examination procedures, and data confidentiality measures, and provided informed verbal consent.

### 2.2 Participant Recruitment and Selection

The inclusion criterion is explicitly stated as: “Non‐pregnant adult women aged ≥ 20 years who provided informed consent. Exclusion criteria: Women with known syndromic heritable connective tissue disorders (e.g., Marfan syndrome, vascular Ehlers–Danlos syndrome, osteogenesis imperfecta, or diagnosed hEDS); active pelvic or abdominal malignancy; active pregnancy; prior hysterectomy or pelvic reconstructive surgery; and incomplete clinical records or uncompleted physical examination.

### 2.3 Participant Flow and Missing Data Handling

Between November 2021 and April 2024, a total of 885 women presenting to the outpatient gynecology clinic were assessed for study eligibility. Of these, 73 women were excluded based on predefined criteria: 28 due to prior hysterectomy or pelvic reconstructive surgery; 18 due to active pregnancy; 9 due to pelvic or abdominal malignancy; 4 due to diagnosed syndromic heritable connective tissue disorders (e.g., Marfan syndrome, vascular Ehlers–Danlos syndrome); and 14 due to incomplete clinical or demographic records. Missing data across primary exposure (Beighton score) and outcome (POP‐Q stage) variables were <1.7% prior to filtration. Complete‐case analysis was implemented for all multivariable models, yielding a final analytical sample of *N* = 812 women (444 POP cases and 368 non‐POP controls) with complete data across all evaluated variables. Missing data were assumed to be Missing Completely at Random (MCAR), given the low missingness rate and uniform clinical documentation procedures.

### 2.4 Control Group Selection and Mitigation of Selection Bias

To minimize selection bias inherent in tertiary care hospital settings, non‐POP control participants (*n* = 368) were concurrently recruited from women presenting to the same outpatient clinic during the study period for non‐prolapse‐related conditions (e.g., cervical cancer screening, routine health examinations, benign ovarian cysts, or physiological discharge). All control participants underwent identical physical examinations and were confirmed to have no clinical evidence of pelvic organ prolapse (POP‐Q Stage 0).

## 3 Clinical Assessment and Outcome Measurement

Pelvic Organ Prolapse (POP): Clinical evaluation of pelvic organ support was performed by senior gynecologists using the standardized Pelvic Organ Prolapse Quantification (POP‐Q) system [10]. Physical examination was performed in the dorsal lithotomy position with the patient performing maximum strain (Valsalva maneuver). Prolapse was staged from Stage 0 to Stage IV according to standard criteria based on the supreme distal aspect of the prolapsed segment relative to the hymenal ring: Stage 0: No prolapse demonstrated. Stage I: Supreme distal point of prolapse is >1 cm above the level of the hymen. Stage II: Supreme distal point of prolapse is ≤1 cm proximal or distal to the hymenal plane. Stage III: Supreme distal point of prolapse is >1 cm below the hymenal plane but protrudes no more than 2 cm less than total vaginal length. Stage IV: Complete eversion of the overall length of the lower genital tract.

For binary analytic comparisons, cases were defined as women with POP‐Q Stage I or higher (*n* = 444), and controls were defined as women with POP‐Q Stage 0 (*n* = 368). To evaluate prolapse severity trends while accounting for lower frequencies in advanced stages (Stages III and IV), POP‐Q severity was grouped into three analytical categories: Stage 0 (no prolapse; *n* = 368), Stage 1 (Stage I prolapse; *n* = 196), and Stage 2+ (combined Stages II, III, and IV; *n* = 248). Grouping Stages II–IV ensured balanced subgroup numbers for ordinal analysis and visualization

Generalized Joint Hypermobility (JHM) Assessment: Generalized JHM was assessed using the 9‐point Beighton scoring system, a validated tool for evaluating joint laxity [9, 11], performed by a trained physician before pelvic examination to maintain the blinding of the examiner, when possible. The usual 9‐point system scores five tests: dorsiflexion of the fifth metacarpophalangeal joint beyond 90° (1 point for left, 1 point for right); passive opposition of the thumb to the flexor surface of the forearm (1 point for left, 1 point for right); hyperextension of the elbow more than 10° (1 point for left, 1 point for right); hyperextension of the knee more than 10° (1 point for left, 1 point for right); and forward trunk flexion with knees straight, placing hands on the floor (1 point).

According to the study protocol, generalized joint hypermobility was defined exclusively using the primary threshold of a Beighton score ≥ 4/9 [11]. Alternative cutoffs and sensitivity analyses were not evaluated in this study [12, 13].

### 3.1 Assessment of Covariates

Trained investigators collected demographics and clinical covariates through structured clinical interviews and medical record review. Covariate measurements included: (1) age and anthropometrics: continuous age (years) was recorded for all eligible participants aged ≥ 20 years (mean 38.00 ± 8.25 years; range 20–60 years).

Height (m) and weight (kg) were measured using digital scales and stadiometers. BMI was calculated based on weight (kg) divided by the square of height (m) (kg/m^2^). (2) Obstetric history and delivery type: Gravida, parity, abortion count, place of delivery (at home/hospital), and difficulty of delivery (easy/difficult) were systematically recorded. Delivery type classification was done into four categories: vaginal delivery alone, cesarean delivery alone, vaginal and cesarean delivery, and nulliparity. (3) Behavioral, familial, and clinical correlates: Heavy lifting was evaluated via self‐reported structured clinical interviews and defined as regular manual lifting of loads weighing >10 kg on a daily basis (≥ 3 times/day for a cumulative duration of ≥ 1 year) during occupational, agricultural, or routine domestic tasks (e.g., carrying water, heavy laundry, or dependents). This >10‐kg threshold was adopted from established epidemiological and occupational pelvic floor studies [14, 15], while reflecting common physical burden patterns documented in regional low‐ and middle‐income country populations [16] and family history of pelvic organ prolapse (defined as first‐degree female relatives). Other secondary clinical covariates recorded as binary variables included a history of chronic cough (daily coughing >8 weeks) and chronic straining while defecating. (4) History of hysterectomy and reconstructive pelvic surgery: Women with a history of prior hysterectomy or pelvic reconstructive surgery were excluded to prevent surgical anatomical alterations from confounding native connective tissue laxity assessments. (5) Age as a biological proxy: Menopausal status and hormonal parameters were not directly measured. Continuous age (years) was included in all multivariable models as a surrogate for age‐related tissue remodeling. However, using age as a proxy cannot fully account for individual variability in menopausal timing or hypoestrogenic states, leaving open the possibility of residual confounding.

### 3.2 Statistical Analysis

Statistics were presented in accordance with the Guidelines for Reporting of Statistics for Clinical Research in Urology by Assel et al., 2018 [17] and the SAMPL guidelines. Normality of the continuous variables' distribution was evaluated visually and using the Shapiro–Wilk test. Continuous variables are presented as mean ± SD, and categorical variables as *n* and %. Differences between POP cases and controls were analyzed in the following way: for continuous variables, by using the Welch's two‐sample *t*‐test (due to differences in variance) or the Mann–Whitney U‐test; for categorical variables, by applying Pearson's *χ* ^2^ test of independence (*χ* ^2^).

Univariate odds ratios (ORs) with 95% confidence intervals (CIs) were calculated based on 2 × 2 contingency tables. To evaluate independent predictors of pelvic organ, prolapse (POP status: case = 1, control = 0), a single comprehensive multivariable logistic regression model was executed (*N* = 812). Covariates simultaneously entered into the model included JHM status (binary, Beighton score ≥ 4/9), age (years), body mass index (BMI, kg/m^2^), parity (continuous), heavy lifting (yes/no), family history of POP (yes/no), delivery mode (categorical: exclusive vaginal delivery [reference], exclusive Cesarean section, combined vaginal and Cesarean section, and nulliparous), chronic cough (yes/no), and chronic straining during defecation (yes/no).

Model diagnostic procedures included evaluating multicollinearity via Variance Inflation Factors (VIF < 2.5 for all covariates) and assessing for model misfit using the Hosmer–Lemeshow test. Influential observations were evaluated using Cook's distance, standardized leverage values, and DFBETAs to ensure stability of the parameter estimates.

*p* values for all tests were calculated using two‐tailed tests with a significance level *α* = 0.05. *p* values for all test results are provided according to established guidelines (*p* < 0.001; *p* values ranging from 0.001 to 0.01 given to the third decimal; *p* values greater than 0.01 are given to the second decimal). Data processing was done via Python 3.10 and R 4.2.1.

## 4 Results

### 4.1 Participant Characteristics

Out of 885 women screened for study inclusion, 812 met all eligibility criteria and provided complete data for primary complete‐case analysis (Figure 1). The final sample comprised 444 POP cases (54.7%, POP‐Q Stage I–IV) and 368 non‐POP controls (45.3%, POP‐Q Stage 0).

**Figure 1:**
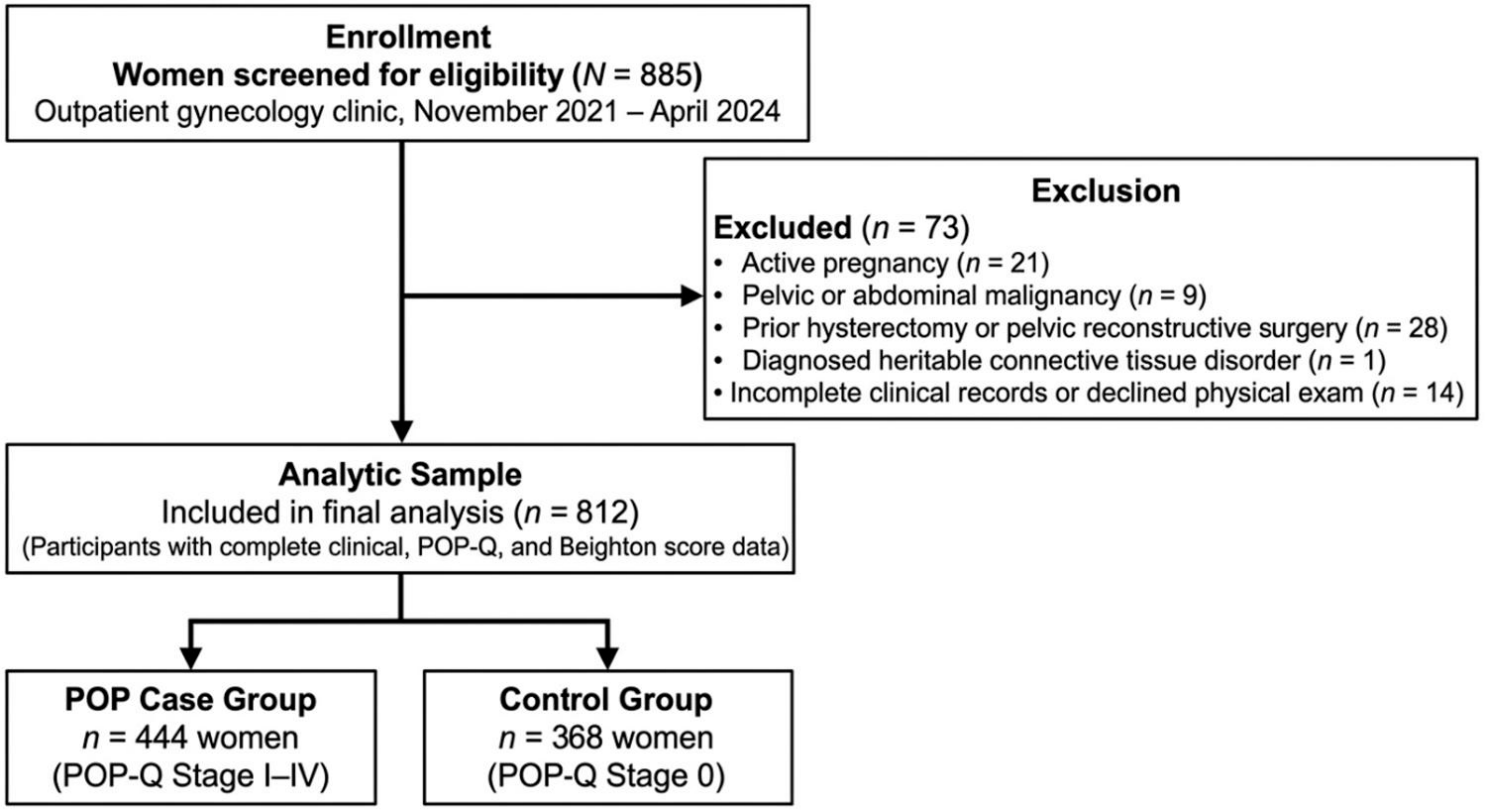
Participant flow diagram (STROBE framework).

Baseline demographic and clinical characteristics are summarized in Table 1. Women with POP were slightly older (mean 39.05 ± 8.58 years vs. 36.73 ± 7.67 years; Welch's *t* = 4.07, *df* = 805.4, *p* < 0.001) and had higher average parity (5.77 ± 3.04 births vs. 4.14 ± 3.21 births; Welch's *t* = 7.38, df = 765.2, *p* < 0.001) and gravidity (6.91 ± 3.39 vs. 5.04 ± 3.89; Welch's *t* = 7.22, df = 733.6, *p* < 0.001) compared to controls. Mean BMI was marginally higher in the POP group (28.34 ± 5.02 kg/m^2^ vs. 27.51 ± 6.21 kg/m^2^, Welch's *t* = 2.07, df = 701.7, *p* = 0.039).

**Table 1:** Baseline demographic and clinical characteristics of participants (*N* = 812).

Variable	POP cases (*n* = 444)	Controls (*n* = 368)	Statistical test	Statistic (df)	*p* value
Age (years), mean ± SD	39.05 ± 8.58	36.73 ± 7.67	Welch's *t*‐test	*t* = 4.07 (805.4)	< 0.001
BMI (kg/m^2^), mean ± SD	28.34 ± 5.02	27.51 ± 6.21	Welch's *t‐*test	*t* = 2.07 (701.7)	0.039
Parity, mean ± SD	5.77 ± 3.04	4.14 ± 3.21	Welch's *t‐*test	*t* = 7.38 (765.2)	< 0.001
Gravida, mean ± SD	6.91 ± 3.39	5.04 ± 3.89	Welch's *t*‐test	*t* = 7.22 (733.6)	< 0.001
Joint hypermobility, *n* (%)	300 (67.6%)	40 (10.9%)	Pearson's *χ* ^2^	*χ* ^2^ = 265.77 (1)	< 0.001

*Note:* Joint hypermobility defined as Beighton score ≥ 4/9. *p* values derived from two‐sided statistical tests.

Abbreviations: BMI = body mass index, POP = pelvic organ prolapse, SD = standard deviation.

### 4.2 Unadjusted Association Between Joint Hypermobility and Pelvic Organ Prolapse

Generalized joint hypermobility (Beighton score ≥ 4/9) was present in 67.6% (300/444) of women in the POP group compared with 10.9% (40/368) in the control group (Pearson's *χ* ^2^ = 265.77, df = 1, *p* < 0.001). This corresponds to an unadjusted odds ratio of 17.08 (95% CI: 11.64–25.07). The proportion of JHM between the two groups is illustrated in Figure 2.

**Figure 2:**
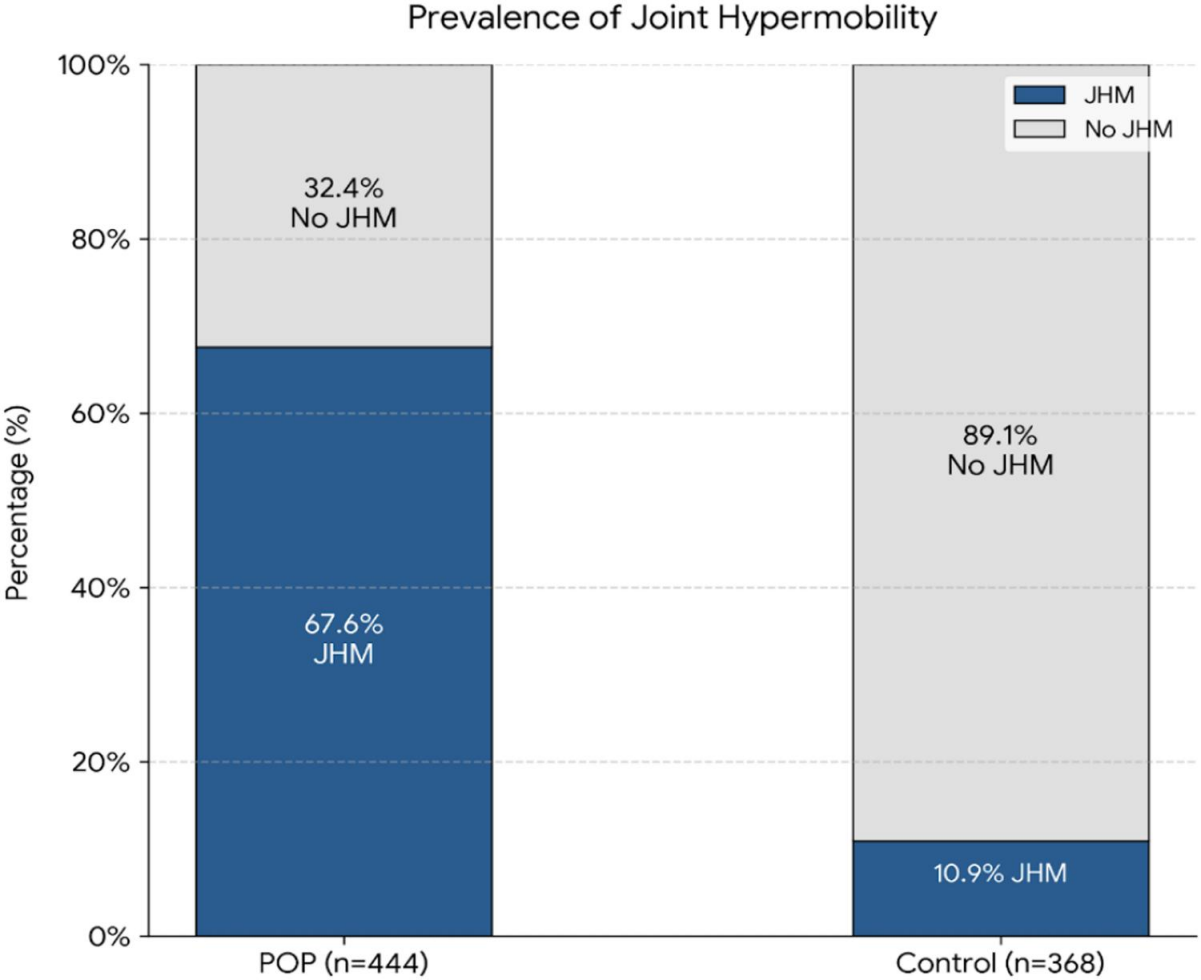
Prevalence of generalized joint hypermobility (JHM, Beighton score ≥ 4) among women with pelvic organ prolapse (POP) versus non‐POP controls (*p *< 0.001).

### 4.3 Multivariable Logistic Regression Analysis

In the comprehensive multivariable logistic regression model incorporating all demographic, clinical, obstetric, and behavioral covariates simultaneously (*N *= 812; Table 2), generalized JHM remained strongly and independently associated with POP (aOR 28.11, 95% CI: 17.21–45.91; Wald *χ* ^2^ = 177.76, *p *< 0.001). Family history of POP (aOR 9.69, 95% CI: 5.58–16.83; Wald *χ* ^2^ = 65.01, *p *< 0.001), heavy manual lifting (aOR 6.48, 95% CI: 4.05–10.37; Wald *χ* ^2^ = 60.76, *p *< 0.001), and parity per individual birth (aOR 1.17, 95% CI: 1.08–1.27; Wald *χ* ^2^ = 14.16, *p *< 0.001) were also independently associated with increased odds of POP. Age (aOR 1.00, 95% CI: 0.97–1.04; *p *= 0.921) and BMI (aOR 1.00, 95% CI: 0.96–1.04; *p *= 0.998) were not independently associated with POP after multivariable adjustment.

**Table 2:** Multivariable logistic regression analysis of factors associated with pelvic organ prolapse (*N* = 812).

Variable	Adjusted odds ratio (aOR)	95% Confidence interval	Wald *χ* ^2^ statistic	df	*p* value
Joint hypermobility (Beighton ≥ 4)	28.11	17.21–45.91	177.76	1	< 0.001
Family history of POP (yes vs. no)	9.69	5.58–16.83	65.01	1	< 0.001
Heavy lifting (yes vs. no)	6.48	4.05–10.37	60.76	1	< 0.001
Delivery mode (reference: vaginal only)	1	Reference	—	—	—
Nulliparous	0.012	0.002–0.10	16.98	1	< 0.001
Cesarean section (CS)	0.57	0.18–1.83	0.897	1	0.343
Both vaginal and CS	1.05	0.66–1.69	0.048	1	0.826
Parity (per single birth increase)	1.17	1.08–1.27	14.16	1	< 0.001
Age (per single year increase)	1.00	0.97–1.04	0.01	1	0.921
BMI (per 1 kg/m^2^ increase)	1.00	0.96–1.04	0.00	1	0.998
Chronic cough (yes vs. no)	1.35	0.77–2.35	1.086	1	0.297
Straining during defecation (yes vs. no)	0.81	0.50–1.32	0.712	1	0.399

*Note:* Outcome variable: POP status (case = 1, control = 0). Hosmer–Lemeshow goodness‐of‐fit *p* = 0.524.

Abbreviations: aOR = adjusted odds ratio, BMI = body mass index, CI = confidence interval, df = degrees of freedom, POP = pelvic organ prolapse.

Multicollinearity diagnostics demonstrated low variance inflation factors (VIF < 1.4 for all model terms). The Hosmer–Lemeshow goodness‐of‐fit test yielded *χ* ^2 ^= 7.12 (df = 8, *p *= 0.524), providing no statistically significant evidence of lack of fit. Post‐estimation influence diagnostics showed low Cook's distance values (all Cook's *D* < 0.10), while the maximum absolute DFBETA for the joint hypermobility coefficient was <0.12. Overall, the influence diagnostics did not identify evidence of a single observation exerting an extreme influence on the fitted model.

### 4.4 Delivery Mode Dynamics and Additional Clinical Covariates

The subgroup with women giving birth exclusively through vaginal birth was the most common mode of delivery and accounted for 64.2% (*n* = 521/812) of the total sample population in this study. Among this group, 325 women (62.4%) had POP, while 196 (37.6%) were non‐POP controls (POP‐Q Stage 0). JHM was observed in 48.0% (*n* = 250/521) of women with exclusive vaginal deliveries. Within this descriptive subgroup analysis, 93.6% (234/250) of hypermobile women who had delivered vaginally were diagnosed with POP. This finding reflects a high observed co‐occurrence of joint hypermobility and pelvic organ prolapse among vaginally parous women, though formal statistical interaction testing was not performed to confirm effect modification.

Within the same multivariable model, delivery mode categories were evaluated relative to exclusive vaginal delivery. Nulliparous women demonstrated significantly lower odds of prolapse (aOR 0.012, 95% CI: 0.002–0.10; *p *< 0.001). Odds of POP did not significantly differ from exclusive vaginal delivery for women with exclusive Cesarean section delivery (aOR 0.57, 95% CI: 0.18–1.83; *p *= 0.343) or combined vaginal and Cesarean deliveries (aOR 1.05, 95% CI: 0.66–1.69; *p *= 0.826). Secondary non‐obstetric clinical covariates, including chronic cough (aOR 1.35, 95% CI: 0.77–2.35; *p *= 0.297) and straining during defecation (aOR 0.81, 95% CI: 0.50–1.32; *p *= 0.399), were not significantly associated with POP in the fully adjusted model.

The adjusted odds ratios and corresponding 95% confidence intervals are displayed in Figure 3.

**Figure 3:**
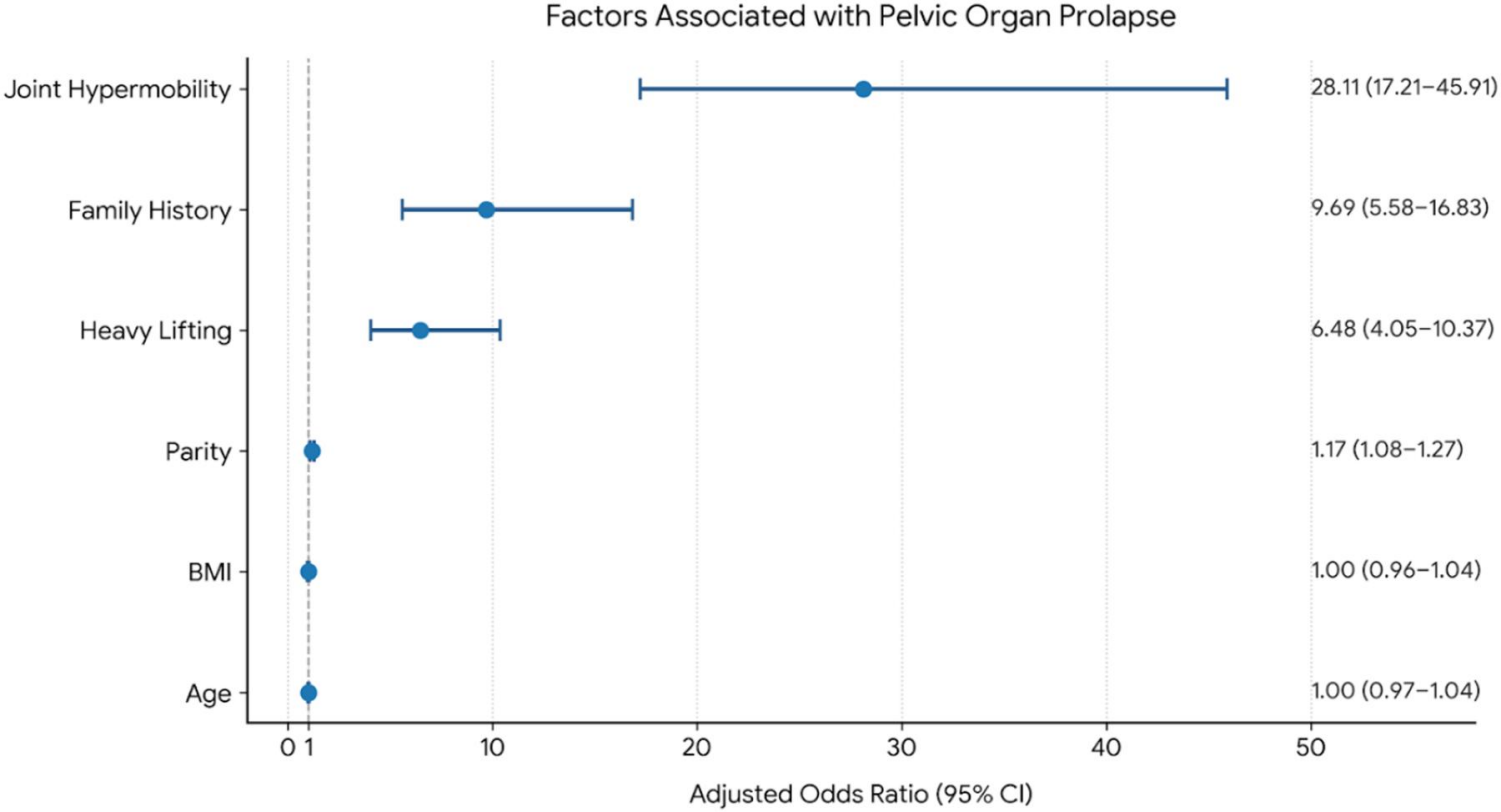
Forest plot depicting multivariable adjusted odds ratios (aOR) and 95% confidence intervals for clinical correlates of pelvic organ prolapse.

### 4.5 Beighton Score Distribution and Prolapse Severity

Distributions of Beighton scores, calculated using kernel density, appeared skewed right among patients suffering from POP as compared to those among controls (Figure 4). There was a high concentration of Beighton scores at 0 and 1 among controls; however, there was a wider distribution among cases, including a high frequency of Beighton scores varying from 3 to 6.

**Figure 4:**
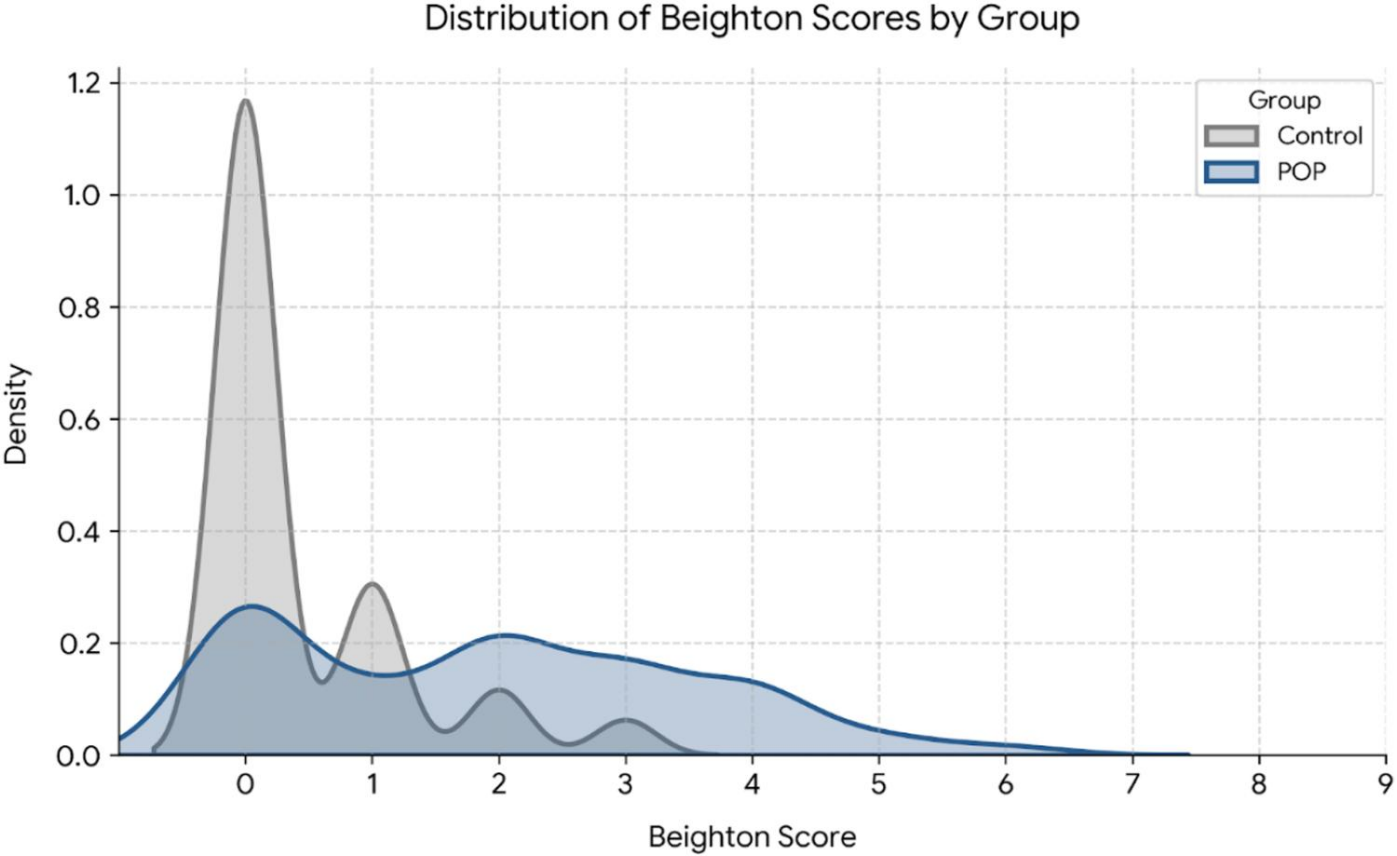
Kernel density distribution of Beighton scores (0–9) in women with pelvic organ prolapse (POP) versus non‐POP controls.

Across the overall cohort (*N* = 812), participant distribution across individual POP‐Q stages was as follows: Stage 0 (*n* = 368, 45.3%), Stage I (*n* = 196, 24.1%), Stage II (*n* = 200, 24.6%), Stage III (*n* = 40, 4.9%), and Stage IV (*n* = 8, 1.0%). For severity trend analyses, participants were evaluated across three collapsed categories: Stage 0 (*n* = 368), Stage 1 (*n* = 196), and Stage 2+ (*n* = 248, combining Stages II–IV).

Beighton score across prolapse severity categories: Median Beighton scores demonstrated a step‐wise increase across advancing prolapse severity categories (Figure 5). Median (IQR) Beighton scores were 0 (0–2) in Stage 0, 2 (0–4) in Stage 1, and 4 (2–6) in Stage 2+ (Kruskal–Wallis test, *p *< 0.001). A statistically significant positive rank correlation was observed between Beighton scores and collapsed POP‐Q severity categories (*ρ* = 0.30, *p *< 0.001).

**Figure 5:**
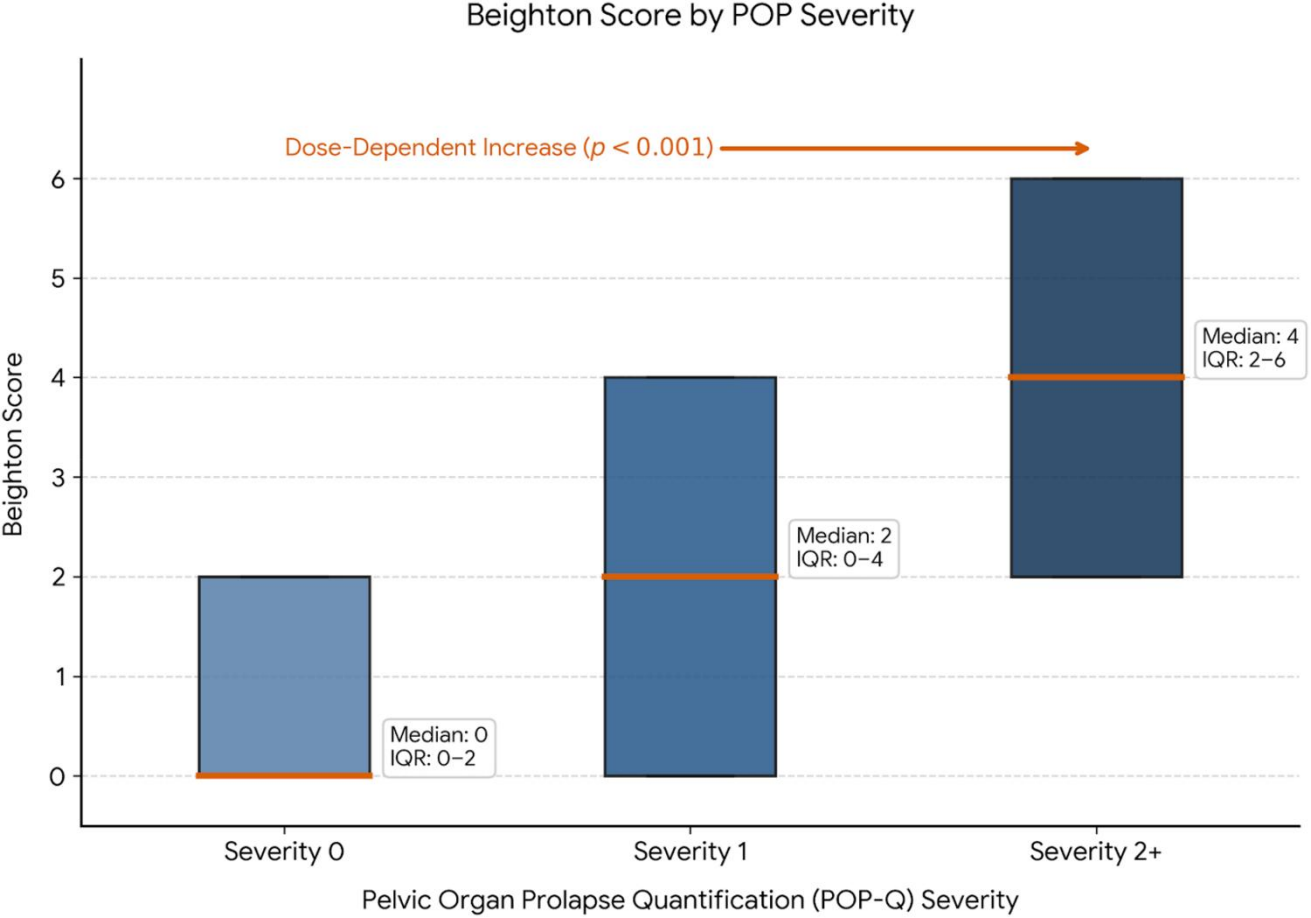
Boxplot distribution of Beighton scores across Pelvic Organ Prolapse Quantification (POP‐Q) severity categories, illustrating higher Beighton score distributions across advancing POP‐Q severity stages. Interquartile range (IQR).

## 5 Discussion

### 5.1 Main Findings

In this hospital‐based cross‐sectional study among 812 Middle Eastern women, generalized joint hypermobility (JHM) showed a statistically significant independent relationship with pelvic organ prolapse. The odds ratio of JHM among women who had POP compared to those without POP was almost 17 times that of control subjects (unadjusted OR). With adjustments for age, BMI, number of pregnancies, delivery type, and parity, joint hypermobility still remained significantly associated (adjusted odds ratio [aOR] 28.11; 95% CI: 17.21–45.91).

Additionally, higher Beighton scores correlated with advancing POP‐Q severity stages.

### 5.2 Biological Mechanisms and Hypotheses

While the lack of longitudinal nature does not allow making any causal conclusions in the current study, the found connection between joint hypermobility and prolapse appears plausible and is corroborated by the evidence accumulated in the connective tissue literature [4, 5, 6]. Proper support of pelvic organs significantly relies upon the passive mechanical features, including viscoelasticity, ensured by collagen and elastin of the endopelvic fascia and ligamentous tissue.

Joint hypermobility is widely acknowledged to be a characteristic marker of the systematic nature of the connective tissue variation [7, 8]. Histological and biochemical analysis of hypermobile connective tissues showed the variations in the ratio of collagen I/III, fibrils diameter distribution, abnormalities in the cross‐linking processes, and alteration in matrix metalloproteinases (MMPs) particularly (MMP‐2 and MMP‐9) and tissue inhibitors of metalloproteinases (TIMPs) expression, respectively [4, 6]. In addition to that, there was the evidence about the disturbances in the pathway of transforming growth factor‐beta (TGF‐β) regulation, being involved in both conditions [6, 7].

Hypothetically, these reported biochemical alterations may decrease the structural stiffness and load‐bearing capacity of pelvic ligaments. Under recurrent intra‐abdominal mechanical strain—such as parturition, physical work, or chronic straining—such underlying tissue vulnerabilities are hypothesized to facilitate progressive connective tissue stretching and subsequent descent of pelvic organs, though longitudinal biological studies are required to confirm this pathway.

### 5.3 Comparison With Existing Literature

Our findings align with previous systematic reviews and meta‐analyses demonstrating an elevated prevalence of JHM in women with pelvic floor dysfunction [7, 9]. For example, a meta‐analysis by Veit‐Rubin et al. [7] reported an overall OR of 2.5–4.5 for prolapse among hypermobile individuals. The higher magnitude of effect observed in our cohort (aOR 28.11) warrants careful interpretation.

This high estimate will definitely be due to certain features of both the model and the population studied.

Features of the population studied: This research took place in a specialized hospital where the population showed a high mean parity (cases: 5.77 and controls: 4.14) and regular heavy lifting, which increases the risk of developing connective tissue pathology.

Features of the model: In case‐control models, a considerable separation of groups in 2 × 2 cross‐tabulation (cases: 67.6% of joint hypermobility trait and controls—10.9%) provides us with high point estimates along with wide confidence intervals (17.21–45.91), indicating statistical precision boundaries that should be interpreted alongside clinical context.

Notably, while age and BMI were elevated in crude univariate comparisons, they did not retain independent statistical significance in multivariable modeling. This suggests that in populations with high parity and prevalent manual labor, biological tissue susceptibility (JHM and family history) [7, 18, 19] and direct mechanical strain (parity and heavy lifting) may represent dominant factors in prolapse occurrence [1, 3, 14, 15].

### 5.4 Clinical Implications

The clinical evaluation of joint hypermobility using the Beighton scale is a simple, non‐invasive examination that can be performed during routine gynecological visits. While early identification of hypermobility in younger or nulliparous women might theoretically identify individuals with connective tissue vulnerability, prospective interventional trials are needed to determine whether JHM assessment improves risk stratification.

Whether initiating conservative strategies—such as ergonomic education, weight management, or pelvic floor muscle training—in hypermobile women without prolapse leads to actual risk reduction remains a hypothesis that warrants formal evaluation in prospective longitudinal studies [20, 21].

### 5.5 Study Limitations

The following limitations should be noted while analyzing these results.

Cross‐sectional study design: The measurement of exposure and outcome was conducted at one time point. Therefore, these results only prove statistical correlation but do not confirm any cause‐and‐effect relationship.

Selection bias: Subjects for the research were drawn from one maternity tertiary hospital, that could result in a bias, as such subjects might be more symptomatic than people in the community in general.

Unmeasured confounding: Several clinical and lifestyle factors were not captured in the primary dataset, introducing potential residual confounding. Specifically, menopausal status was not directly assessed, nor were endogenous estrogen levels or systemic/topical estrogen replacement therapy recorded. Furthermore, smoking history (tobacco use) was not collected or adjusted for in the regression models, which represents a limitation given the known associations between smoking, chronic coughing, and connective tissue degradation. Additional unmeasured obstetric factors include specific operative vaginal delivery techniques (vacuum vs. forceps extraction), third‐ or fourth‐degree perineal lacerations, and history of episiotomy.

Age‐stratified cut‐offs: This study relies on a uniform Beighton score cutoff (≥ 4/9) without age‐stratified sensitivity analyses. Because joint laxity naturally declines with age, using a static threshold may lead to under‐classification of hypermobility in older individuals and over‐classification in younger cohorts. Future studies should formally incorporate age‐stratified cutoffs into their analytical frameworks.

## 6 Conclusions

Generalized joint hypermobility is strongly associated with pelvic organ prolapse in this cross‐sectional study of Middle Eastern women, exhibiting a positive correlation with prolapse severity stage. Family history of prolapse, heavy manual lifting, and parity were additional independent clinical correlates. Future prospective studies are needed to evaluate whether integrating joint hypermobility assessment into routine practice can improve risk stratification or inform targeted preventive strategies for pelvic floor health.

## Author Contributions

**Zekra Ali Aziz:** conceptualization, methodology, software, formal analysis, investigation, data curation, writing – original draft, writing – review and editing, project administration.

## Funding

The author has nothing to report.

## Ethics Statement

This study was conducted in accordance with the Declaration of Helsinki and was approved by the Scientific Research Ethics Committee of Erbil Polytechnic University, Erbil, Iraq (Approval No.: 24/0027 HRE).

## Consent

All participants received a full explanation of the study design, objectives, and procedures, and prior to enrollment, verbal informed consent was obtained.

## Conflicts of Interest

The author declares no conflicts of interest.

## Transparency Statement

Zekra Ali Aziz affirms that this manuscript is an accurate, honest, and transparent account of the study being reported; that no significant aspects of the study have been ignored; and that any discrepancies from the study as planned have been explained.

## Data Availability

The datasets generated and analyzed during the current study are not publicly available. Full data supporting the findings of this study are available from the corresponding author Zekra Ali Aziz, zekra.aziz@epu.edu.iq, upon reasonable academic request.
